# The Teaching Design Methods Under Educational Psychology Based on Deep Learning and Artificial Intelligence

**DOI:** 10.3389/fpsyg.2021.711489

**Published:** 2021-10-04

**Authors:** Zewen Wang, Lin Cai, Yahan Chen, Hongming Li, Hanze Jia

**Affiliations:** ^1^Pan Tianshou College of Architecture, Art and Design, Ningbo University, Ningbo, China; ^2^School of Humanities, Shanghai Jiao Tong University, Shanghai, China; ^3^Faculty of Education, Beijing Normal University, Beijing, China; ^4^College of Elementary Education, Capital Normal University, Beijing, China; ^5^The College of Foreign Languages, Inner Mongolia Normal University, Huhhot, China

**Keywords:** teaching design, artificial intelligence, deep learning, educational psychology, satisfaction survey questionnaire, effective return rate

## Abstract

This study aims to evaluate the practical application value of the teaching method under the guidance of educational psychology and artificial intelligence (AI) design, taking the deep learning theory as the basis of teaching design. The research objects of this study involve all the teachers, students, and students' parents of Ningbo Middle School. The questionnaires are developed to survey the changes in the performance of students before and after the implementation of the teaching design and the satisfaction of all teachers, students, and parents to different teaching methods by comparing the two results and the satisfaction ratings. All objects in this study volunteer to participate in the questionnaire survey. The results suggest the following: (1) the effective return rates of the questionnaires to teachers, students, and parents are 97, 99, and 95%, respectively, before implementation; whereas those after implementation are 98, 99, and 99%, respectively. Comparison of the two return results suggests that there was no significant difference statistically (*P* > 0.05). (2) Proportion of scoring results before and after implementation is given as follows: the proportions of levels A, B, C, and D are 35, 40, 15, and 10% before implementation, respectively; while those after implementation are 47, 36, 12, and 5%, respectively. After the implementation, the proportion of level A is obviously higher than that before the implementation, and the proportions of other levels decreased in contrast to those before the implementation, showing statistically obvious differences (*P* < 0.05). (3) The change in the performance of each subject after 1 year implementation is significantly higher than that before the implementation, and the change in the average performance of each subject shows an upward trend. In summary, (1) the comparison on the effective return rate of the satisfaction survey questionnaire proves the feasibility of its scoring results. (2) The comparison of the survey scoring results shows that people are more satisfied with the new educational design teaching method. (3) The comparison of the change in the performance of each subject before and after the implementation indirectly reflects the drawbacks of partial subject education, indicating that the school should pay the same equal attention to every subject. (4) Due to various objective and subjective factors, the results of this study may be different from the actual situation slightly, and its accuracy has to be further explored in the future.

## Introduction

With the rapid development of electronic technology in the society of today, artificial intelligence (AI) technology and deep learning theory have been widely applied in instructional design to respond to the call of educational psychology. With the rapid growth and deep application of electronic science and technology, AI technology is becoming mature, providing a new development way for most fields and leading the better development of this era. AI technology has been applied to all aspects to accelerate the development of countries all over the world, even at the national strategic level of many countries, including the United States, South Korea, Japan, and China (Abbas et al., 2019a,b). The promulgation of a series of national policies on AI technology around the world marks that mankind has entered the era of intelligence. As a new generation of information technology, AI has also been highly valued by educational circles. Therefore, the application of instructional design in this context should be more scientific and reasonable.

To explore the basic law between learning and teaching with scientific methods (Rogoza et al., 2018; Chen, 2019), the State Council of China in July 2017 pointed out that AI education is an important part of the national strategy (Dai et al., 2018). By 2018, the Ministry of Education has issued a series of AI education policies, which is the full opening of the modern education system (Roll and Wylie, 2016). Therefore, education has made great progress in the era of AI. Teaching design refers to the process of reasonably arranging many elements in teaching according to the requirements of curriculum standards and based on the characteristics of educated objects, so as to formulate a scientific and reasonable teaching plan. It mainly includes the reasonable arrangement of teaching objectives, difficulties, methods, steps, and time (Khalil and Elkhider, 2016; Qian et al., 2018; Maqsood et al., 2021). As one of the many branches of applying psychology in the process of education, educational psychology plays a guiding role in learning and teaching. The research of educational psychology is mainly about the teaching psychology of teachers, psychology of students, and the application of educational theory (Wu and Song, 2019). Teachers help students to learn and form a special “interactive system.” The purpose of educational psychology is to ensure the efficient and harmonious operation of the “interactive system” (Wu et al., 2019b). Deep learning theory refers to the learning in which students can understand and absorb the knowledge they have learned, and apply them by analogy to develop their advanced thinking ability (Waldhoer, 2020). Since its publication, this theory has been valued by people all over the world. After 2000, the theory advocated “active, highly participatory, and critical understanding of learning process, including deep participation of advanced thinking and emphasis of learning results” (Abbas et al., 2021; Aqeel et al., 2021), which has been highly recognized by education experts and triggered teaching reform and practice worldwide (Willingham, 2017). Deep learning theory is in line with the development situation of the world and the needs of curriculum reform in China.

To further understand the effect of the combination of AI and instructional design required by modern teaching, this study carries out an instructional design based on educational psychology theory and AI technology under the guidance of deep learning theory and evaluates the application effect of the design through the satisfaction survey of research objects (Su et al., 2021), so as to obtain practical research results.

## Literature Review

1. Foreign-related research
  At present, AI technology and deep learning algorithm have been widely used in various industries of society, including medical treatment, transportation, manufacturing, and education. The AI technology, statistical modeling, and integrated learning are combined and applied to the classroom teaching evaluation, and the results prove the accuracy of teaching evaluation under this mode is higher and shows a good prospect (Guo et al., 2021). Seren and Özcan (2021) also proposed that the main significance of the application of AI in the education industry is to promote the learning adaptability of students, so as to improve the learning enthusiasm of students. In addition, Timms pointed out that the education model combining AI and deep learning is beneficial to enhance the teaching efficiency and increase the participation of students in teaching, which would be widely supported by students in the future (Timms, 2017). According to the developmental situation of AI technology, some experts put forward that the design of intelligent teaching system in the future can not only simulate the learning process of students and provide adaptive cognitive support, but also adaptively establish social relations with learners (Kulikov et al., 2020), and above all show the good developmental prospect of AI in the future.
2. Domestic-related research
  In China, AI technology has been applied in education and there are many pieces of research. In terms of theory, Zhang and others studied the essential characteristics, core technology, and the development trend of AI education (Zhang et al., 2021). In addition, Li and others described in detail the four specific application forms, five typical characteristics, and the integrated development system of AI and education (Li et al., 2020). Some experts also put forward the initial content and implementation plan of relevant teaching courses in view of the application of deep learning theory in higher fields under the background of AI (Chong et al., 2020). Lei studied the application of open-source AI systems in education and discussed the existing problems and future development space in the process of research and practice (Lei et al., 2020). In a word, the specific practical effect needs further research.

## Methods

### Research Objects

All teachers, students, and students' parents of the Ningbo Middle School are selected as the research objects, with a total number of 1,998. The questionnaires are designed to survey the changes in the performance of students before and after the implementation of the teaching design and the satisfaction of all teachers, students, and parents to different teaching methods. The questionnaires are issued two times based on the number of people counted (Walker and Ogan, 2016). They are first distributed before the implementation of the teaching design based on deep learning theory under the precondition of the educational psychology theory and AI technology (Cowie, 2020). They are second distributed after the teaching design is implemented for 1 year, with a total of 2,000 questionnaires each time. For the first distribution, 238 questionnaires are issued to teachers, 230 questionnaires are effectively returned; 881 questionnaires are issued to students and their parents, with effectively returned numbers of 870 and 839, respectively. For the second time, 238 questionnaires are issued to teachers, and 234 questionnaires are effectively returned; 881 questionnaires are issued to students and their parents, respectively, and the numbers of questionnaires effectively returned are 875 and 871, respectively. All objects in this study volunteered to participate in the questionnaire survey. This study has been declared for research ethics and has been approved.

### Representative Teaching Design Models

**Figure 1:**
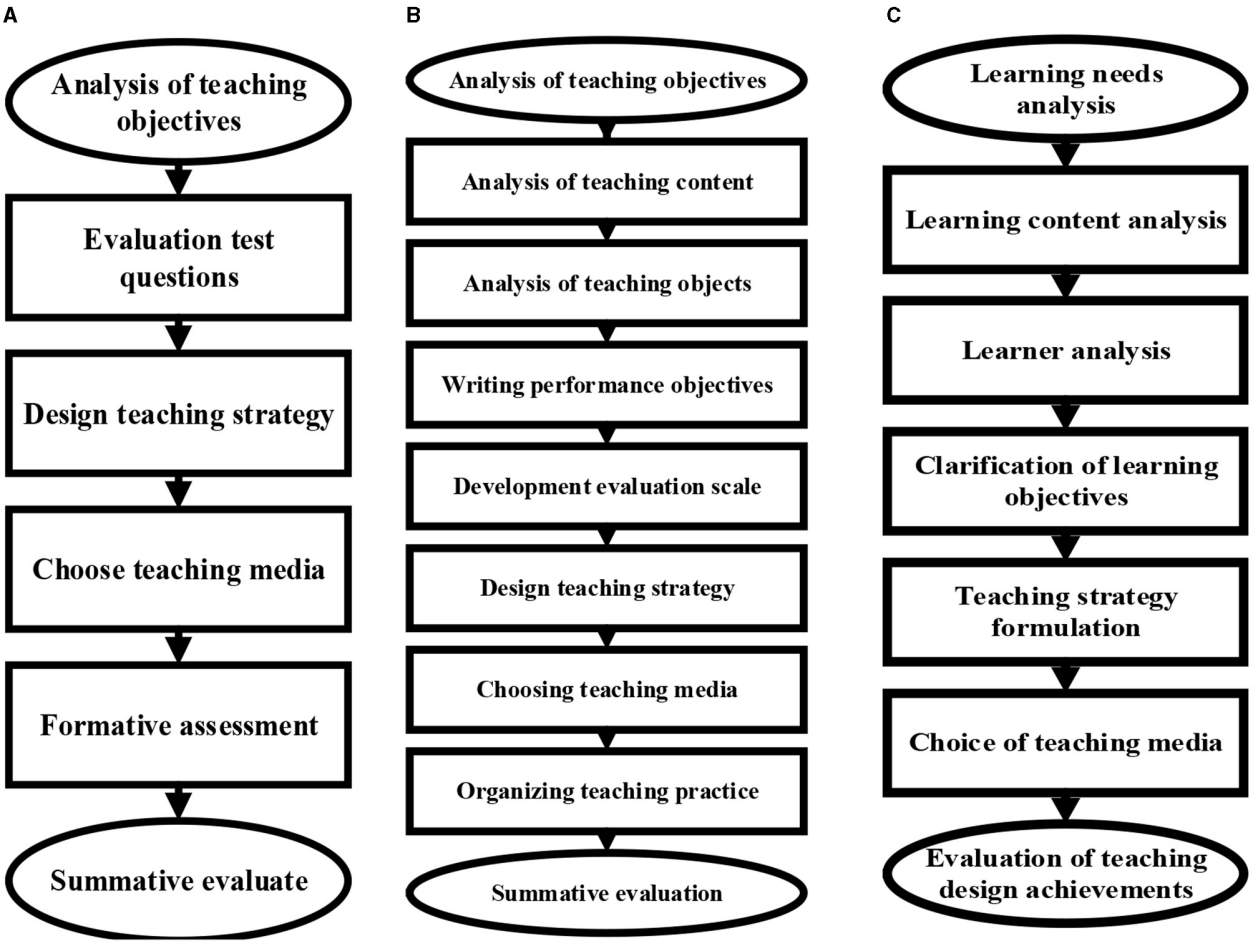
The specific links of representative teaching design models. **(A–C)** showed the links of Gagne's system view teaching design model, Dick Carey's teaching design model, and the general teaching design mode, respectively.

At this stage, the more representative teaching design models include the system view teaching design model proposed by Gagne, the teaching design model proposed by Dick Carey, and the general teaching design mode. Of these, the general model is the most commonly used one (Jabaay et al., 2020; Susilawati and Paidi, 2020). The specific design links of the above three models are shown in Figure 1.

### Multilevel Influencing Factors of Deep Learning Theory-Based Teaching Design Model

**Figure 2:**
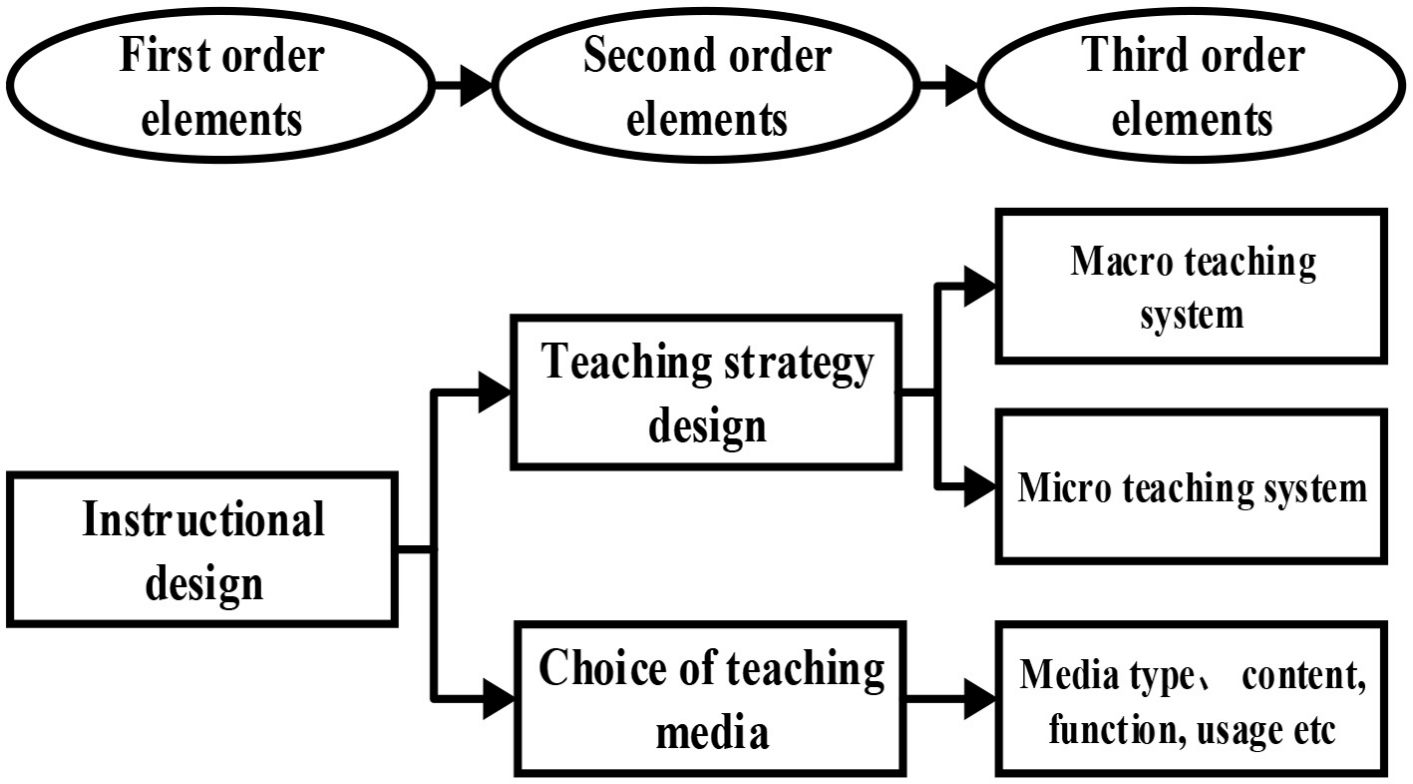
The three-order relationship during the construction of teaching design model based on the deep learning theory.

A third-order deep learning theoretical model can be established based on the systemic principles of deep learning theory. The model is a three-dimensional, multidimensional, open non-linear structure, and is composed of multiple layers and elements. The third-order relationship in the established teaching design model in the deep learning theoretical model (Aderibigbe, 2021) is shown in Figure 2. The teaching design model based on the deep learning theory in this study is also constructed on this basis and applied to actual teaching for verification and evaluation.

### Research Methods

1. **Literature analysis:** The literature is analyzed to collect and sort out the development, current situation, and teaching process based on academic journals and books in educational psychology and AI technology and analyze the existing problems (Shen et al., 2019; Lebni et al., 2020).
2. **Questionnaire survey:** The teaching satisfaction survey questionnaire is improved based on the teaching design in this study (Local Burden of Disease HIV Collaborators, 2021; Su et al., 2021). The contents of the questionnaire for teaching satisfaction mainly include the age, gender, occupation, family economy, education level, and other basic information of surveyed persons, aiming to understand the guiding ideology, basic structure, teaching objectives, analysis of students, and analysis of teaching materials. In addition, it analyzes whether the management methods in the teaching process, the arrangement of homework, the design of examination methods, and teaching strategies are reasonable and scientific (Xiao-Ru et al., 2018). A reasonable evaluation is given based on the degree of changes in the learning situation of the students before and after the implementation of the teaching design. The specific evaluation criteria are shown in Table 1. The reliability and validity of the questionnaire have been tested to enhance the credibility of this study (Ohanian, 1990). Reliability to be verified can be classified into testing reliability and split-half reliability. Eleven items in the questionnaire are divided into six odd-numbered items and five even-numbered items. The sum scores of the odd-numbered item and even-number items are calculated. It is found that the Pearson correlation coefficient of the two is 0.501, which shows obviously statistical significance (*P* < 0.01). Validity verification refers to testing the consistency among the items in the instructional design questionnaire; the total score of the questionnaire is undertaken as the criterion, and the Pearson correlation coefficient between the score of each item and the total score is basically larger than 0.500, indicating that the consistency of each item is better.
3. **Structural equation model:** The satisfaction is evaluated using the structural equation model (SEM) in this paper. SEM is also called path modeling, and it is a statistical method based on statistical analysis techniques to explore macroscopic laws from microscopic individuals (Monecke and Leisch, 2015). The partial least squares (PLS) are adopted to analyze the SEM generally.

The equation that uses mathematical expressions to describe the relationship between measurable variables and hidden variables is called the measurement equation, which can be expressed as follows:


(1) $$\begin{array}{r} \{\begin{array}{c} X_{i}=A_{X}\alpha +\beta \\ Y_{j}=A_{Y}\chi +\delta \end{array} \end{array}$$

In the above equation, *X*_*i*_ and *Y*_*j*_ refer to the column vector formed by the *i*-th and *j*-th measured variables; *A*_*X*_ and *A*_*Y*_ are matrixes; α and χ represent the column vectors, and β and δ refer to the error terms.

**Table 1:** Evaluation on the teaching design (score).

**Evaluation contents**	**Very satisfied**	**Satisfied**	**General**	**Dissatisfied**
Guiding ideology	10	5 ~ 3	2	0 ~ 1
Resource analysis	5	4 ~ 3	2	0 ~ 1
Student analysis	5	4 ~ 3	2	0 ~ 1
Target design	10	5 ~ 3	2	0 ~ 1
Design structure	25	9 ~ 15	4 ~ 8	0 ~ 3
Teaching strategy	5	4 ~ 3	2	0 ~ 1
Homework design	5	4 ~ 3	2	0 ~ 1
Analysis on teaching materials	5	4 ~ 3	2	0 ~ 1
Management method	10	5 ~ 3	2	0 ~ 1
Examination method	5	4 ~ 3	2	0 ~ 1
Design Features	15	6 ~ 8	3 ~ 5	0 ~ 2

*Grade A: 90–95 scores; grade B: 85–90 scores; grade C: 80–85 scores; D: lower than 80 scores.

Then, the relationship between the exogenous hidden variables and the endogenous hidden variables in the structural equation is as follows:


(2) $$\begin{array}{r} \chi =B\chi +ℑ\alpha +\gamma \end{array}$$

In equation (2) above, *B* refers to the relationship between various endogenous hidden variables; ℑ is a matrix, indicating the influence of exogenous hidden variables on endogenous hidden variables; and γ represents the residual item.

The calculating steps of PLS are given as follows:

First, the expression of the hidden variable is obtained using iteration:


(3) $$\begin{array}{r} \bar{\alpha_{j}}=\sum \phi_{jh}x_{jh} \end{array}$$

The above equation is the linear combination of manifest variable *x* based on weighting φ.

Second, the common PLS method is adopted to obtain the estimation of the coefficients of internal relations and external relations, that is, to obtain a measurement of the relationship between hidden variables and the measurable variables.

Finally, various values should be obtained. Since there is the same estimated value for each hidden variable to be the number of observations, which is assumed to be *m*, the estimated ${\bar{\alpha }}_{j}$ (*j* = 1,...,*m*) can be obtained. Based on these estimated values, the survey satisfaction index can be calculated with the following equation:


(4) $$\begin{array}{r} \bar{\alpha }=\frac{\frac{1}{m}\overset{m}{\underset{j}{\sum }}{\bar{\alpha }}_{j}-\underset{j}{\min}\left({\bar{\alpha }}_{j}\right)}{\underset{j}{\max}{\bar{\alpha }}_{j}-\underset{j}{\min}{\bar{\alpha }}_{j}}\times 100\% \end{array}$$

**4 Analysis of the performance of students:** The average performances in the main subjects (including Chinese, mathematics, English, physics, chemistry, biology, politics, and history) of the whole school students 1 year ago and 1 year later are sorted out for comparative analysis.

### Statistical Analysis

SPSS22.0 statistical software is used for data analysis, the measurement data is expressed as mean ± SD ($\bar{x}$ ± s), and measurement data among groups are compared by *t*-test. The count data is expressed in the form of a percentage (%) and was compared using the χ^2^ test among groups. The k-s test is used to test whether the sample conforms to the normal distribution. The data conforming to the normal distribution is tested by the *t*-test, and the Kruskal–Wallis H and F-test are used for the data that does not conform to the normal distribution. *P* < 0.05 indicates that the difference is statistically significant.

## Results

### Comparison of the Return Rate of the Questionnaire Survey Before and After the Implementation of the New Teaching Design

In this study, satisfaction with the teaching design is surveyed based on deep learning theory before its implementation and after its implementation for 1 year. In addition, the survey questionnaire is returned. For the first distribution, 238 questionnaires are issued to teachers, and 230 questionnaires are effectively returned; 881 questionnaires are issued to students and their parents, and 870 and 839 are effectively returned, respectively. Therefore, the effective return rates are 97%, 99%, and 95%, respectively. For the second time, 238 questionnaires are issued to teachers, and 234 questionnaires are effectively returned; 881 questionnaires each are issued to students and their parents, and 875 and 871 are returned effectively, respectively. Therefore, the effective return rates are 98, 99, and 99%, respectively. The effective return rates from teachers, students, and parents of the two times distributions of questionnaires are compared, and the results show that the effective return rate from teachers differs by 1%, that from students does not change, and that from parents differs by 4%. Therefore, the change of the effective return rate between the two distributions is relatively small, showing no statistical significance (*P* > 0.05), proving the feasibility of comparing the results of the two questionnaire surveys.

### The Age, Gender, and the Educational Background Distribution of All Filled-In Persons

**Figure 3:**
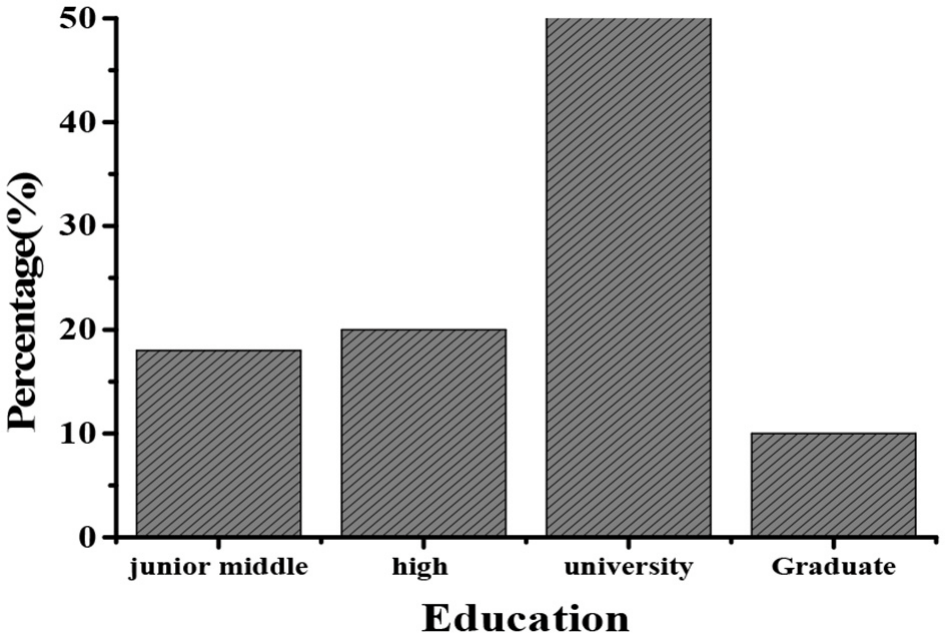
Distribution on the education level of subjects.

According to the basic information filled in all the satisfaction survey questionnaires returned, statistics are collected based on the gender, age, and education level of the filled-in persons. The specific information is shown in Table 2. There are about 59% of men and 41% of women; there are 912 people aged 10–15 years old (45%), 1,054 people aged 25–45 years old (53%), and 34 people over 45 years old (2%). For the distribution of education level, 18% of the subjects with a junior high school degree or below, 20% of the subjects with a high school degree, 52% of the subjects with a University degree, and 10% of the subjects with a graduate degree or above (as shown in Figure 3) were selected. According to the above statistics, there is no obvious difference in gender, which has little effect on the survey results, but the unevenness of age and education will have a larger impact on the survey. This is a point to be analyzed in the subsequent analysis.

**Table 2:** Proportions of filled-in persons in gender and age.

	**Indicator**	**Proportion (%)**
Gender	Male	59
	Female	41
Age (years old)	10–15	45
	25–45	53
	>45	2

### Results Analysis of Related Literature Surveys

**Figure 4:**
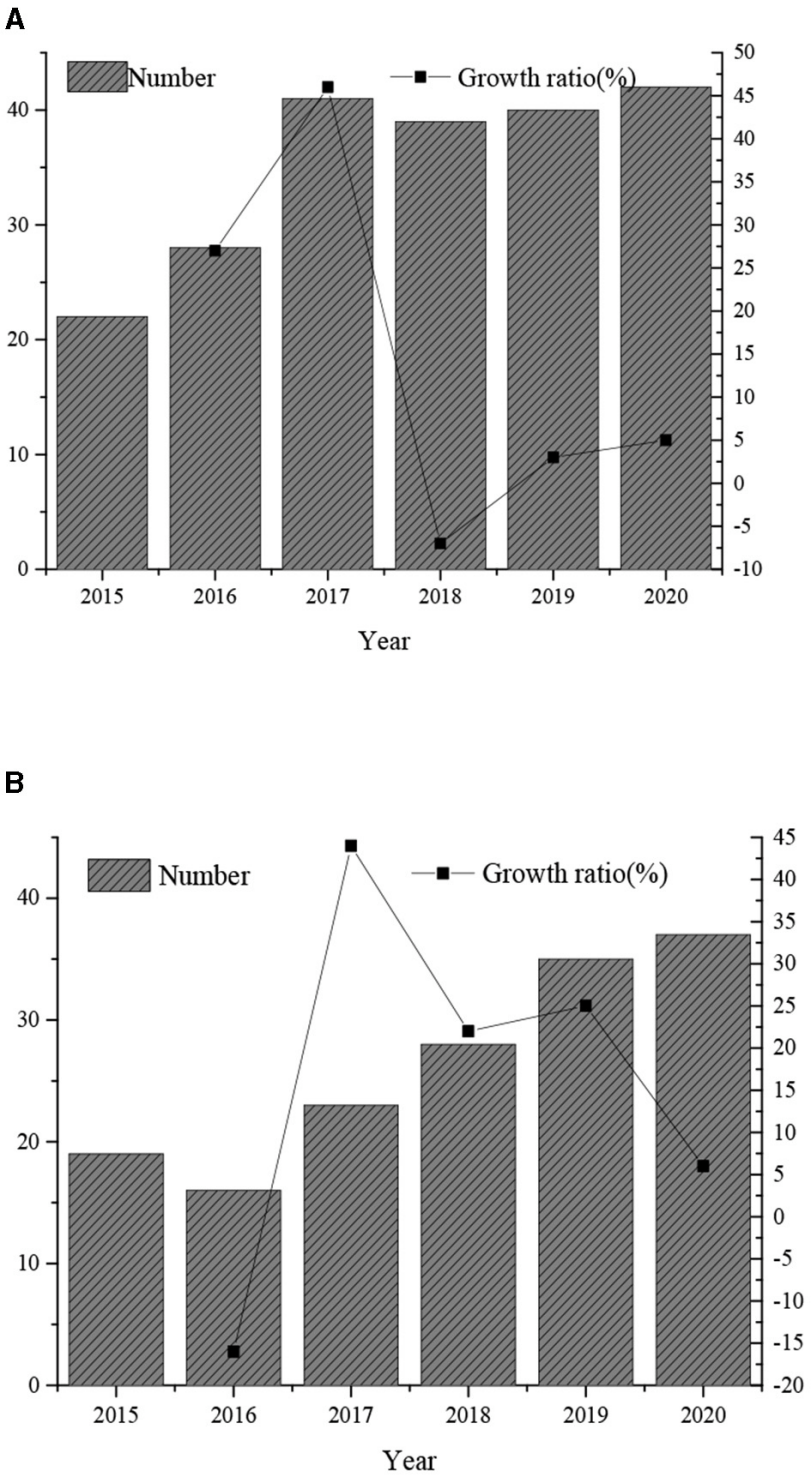
Statistics of related literature. **(A,B)** show the statistics results of literature related to AI and deep learning, respectively.

As illustrated in Figure 4, there is more and more research literature studying the teaching design based on AI and deep learning in recent years. It shows that AI and instructional design have gradually become the research hotspots.

### Comparison on Satisfactions of Two Surveys

**Figure 5:**
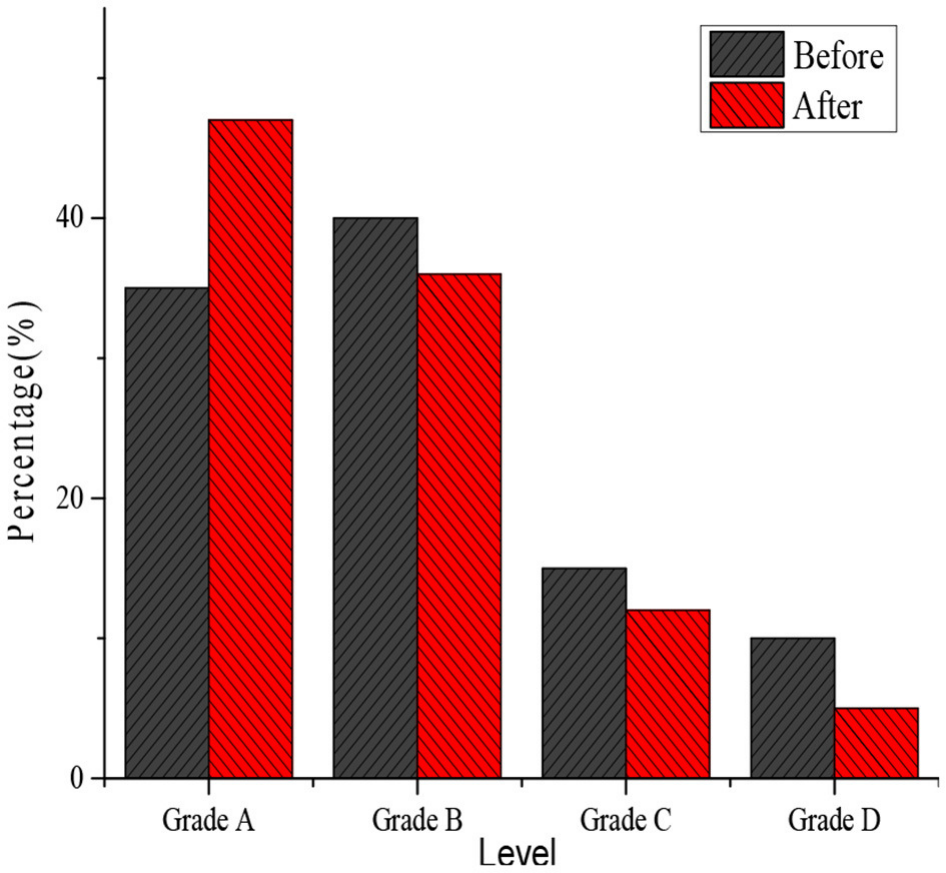
Comparison on satisfactions of two surveys.

Figure 5 shows the statistics of the satisfaction survey questionnaire returned two times. Before the implementation of the new teaching design, the proportions of satisfaction questionnaire scores of levels A, B, C, and D account for 35, 40, 15, and 10%, respectively. After 1 year of implementation, the proportions of satisfaction questionnaire scores of levels A, B, C, and D account for 47, 36, 12, and 5%, respectively. Therefore, the proportion of the level A scores after the implementation is significantly higher than before the implementation, whereas the proportions of the B, C, and D levels are all higher than those after the implementation. Such results indicate that the respondents are more satisfied with the new teaching design, and the comparison shows statistical significance (*P* < 0.05). Such results suggest that optimizing some teaching methods is accepted more by students and parents.

### Comparison on Change in the Performance of Students Before and 1 Year After the Implementation of the New Teaching Model

**Figure 6:**
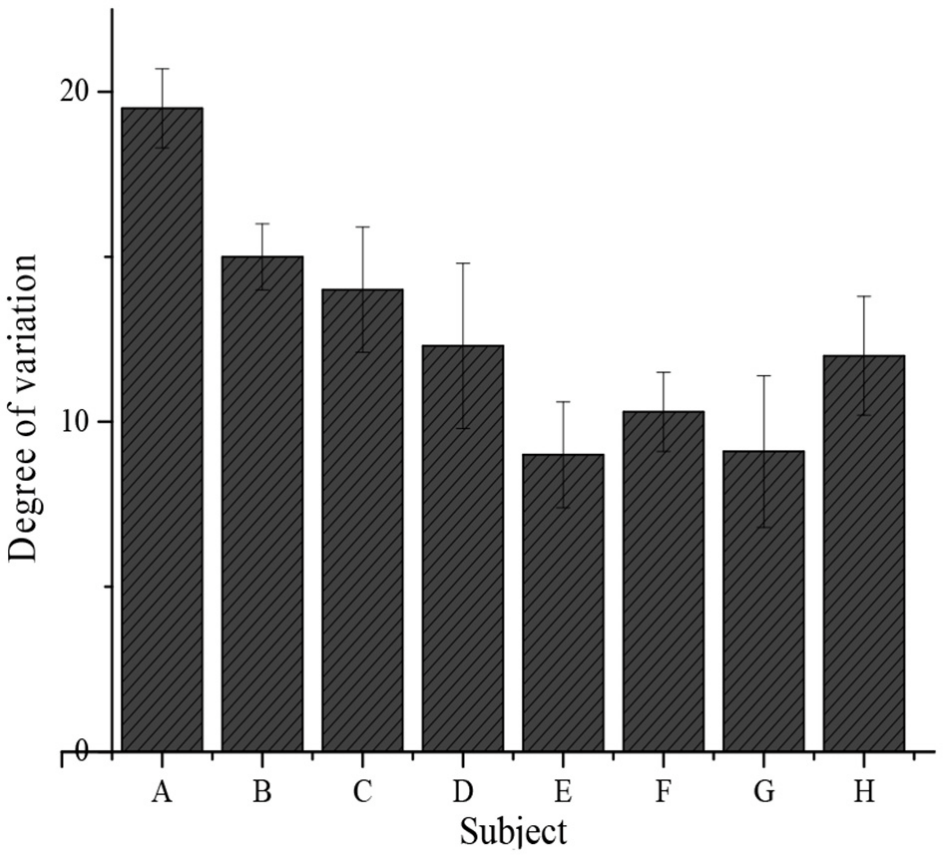
Change in performance of each subject. **(A–H)** refers to mathematics, Chinese, English, physics, chemistry, biology, politics, and history, respectively.

The average scores of each subject of the students of the whole school before and 1 year after the implementation of the new teaching model are collected and compared, as shown in Table 3. After analysis, it is found that the general trend of change in the average score of each subject is increasing. Figure 6 illustrates the change in the performance of each subject. It reveals that three main subjects (mathematics, language, and English) change greatly, indirectly reflecting the shortcomings in education in China that no enough attention is paid to other subjects. In addition, analysis on the relationship between each score in the questionnaire and the score reveals that the higher the scores of student analysis, teaching strategy, and textbook analysis, the higher their scores. The total scores of the above three items are 5, 3.5, and 0.5 in descending order, and the corresponding student scores are 750, 540, and 398 points (100 points per subject). Therefore, there is a proportional trend among them.

**Table 3:** Performance of students before and 1 year after implementation of the new teaching mode (scores).

**Subject**	**Before**	**One year later**
Mathematics	90.2 ± 2.5	109.7 ± 1.9
Chinese	88.2 ± 4.1	103.2 ± 3.4
English	86.2 ± 3.9	100.2 ± 2.3
Physics	77.5 ± 2.1	88.2 ± 2.7
Chemistry	78.2 ± 4.7	89.2 ± 2.9
Biology	79.9 ± 5.2	90.2 ± 3.1
Political	78.1 ± 2.1	87.2 ± 2.4
History	79.2 ± 2.5	91.2 ± 3.1

## Discussion

### Principal Results

In this study, satisfaction with the teaching design is surveyed based on deep learning theory before its implementation and after its implementation for 1 year. The effective return rates from teachers, students, and parents of the two times distributions of questionnaires are compared. The results show that the effective return rates of the questionnaires to teachers, students, and parents are 97, 99, and 95%, respectively, before implementation; whereas those after the implementation of the proposed teaching design are 98, 99, and 99%, respectively. Therefore, there was no obvious difference statistically (*P* > 0.05), so the comparison of the two surveys is feasible. At the same time, the scores before and after implementation of the new teaching design reveal that the proportions of levels A, B, C, and D are 35, 40, 15, and 10% before implementation, respectively; whereas those after implementation are 47, 36, 12, and 5%, respectively. After the implementation, the proportion of level A is obviously higher than that before the implementation, and the proportions of other levels are lower in contrast to those before the implementation, showing statistically obvious differences (*P* < 0.05). It shows that the respondents are more satisfied with the new educational design. The change in the performance of each subject after the new teaching design is implemented for 1 year is significantly higher than that before the implementation, and the average performance of each subject shows an increasing trend. The changes in mathematics, language, and English are the greatest. It is found that the higher the score for student analysis, teaching strategy, and textbook analysis, the higher the score, so there is a proportional trend among them. Such results suggest that it is necessary to fully understand students and be familiar with teaching materials to have good teaching strategies and provide students with better teaching quality (Liu and Chen, 2021).

### Comparison With Prior Works

In this study, related research documents in recent years have been compared statistically, and it is found that the frequency of research on AI combined with teaching is the highest. Many research experts discussed the application of AI technology in teaching. They also pointed out that the integration of education and AI technology can not only improve the teaching efficiency of teachers but also attract students to better integrate into the classroom. Moreover, the system design of AI education in the future is likely to model the learning situation of students, improving the current teaching methods to provide students with a better learning environment (Khazaie et al., 2021; Taufik et al., 2021). Other experts have proposed that traditional learning methods have to be upgraded and transformed with the integration of education and AI, which can not only provide a good learning experience for learners but also provide related researchers with more basis for improvement in learning principles (D'Mello, 2016; Nye, 2016). Some experts have applied AI technology and electronic machinery to the daily study of students for further analysis and discussion, and have achieved good learning results (Wen and Yue, 2021). There are also some related literature collections that discuss the application prospects, challenges, and methods of AI in the education system due to the existence of unbalanced social conditions (Boulay and Luckin, 2016; Pinkwart, 2016; Chen, 2018). In addition, the application of deep learning theory in teaching design has been studied, too. Some researchers have proposed that the main goal of deep learning-based teaching design is to help students master key skills (“6Cs”: character, citizenship, communication, critical thinking and solving, collaboration, and creativity and imagination) (Yang and Juan, 2016). The results of this study also concluded that teaching design in the context of teaching psychology combined with AI technology and deep learning theory is more popular than the previous educational model in terms of teaching process and effect. It is not unreasonable that the educational method in the context of educational psychology will make students more acceptable. Some research experts have proposed that scientific and reasonable teaching activities can be formulated based on the psychological needs and psychological conditions of students in different periods, which can be more effectively stimulate the interest of students in learning (Wu et al., 2019a). There is research on the practice of junior middle school chemistry smart classroom teaching under the background of “AI + education.” It shows that when students accept the smart classroom teaching mode, the learning subjectivity of students is more prominent in the smart classroom, and the learning effect of students is gradually improved in the smart classroom (Sapci and Sapci, 2020). During the teaching, students should not be blindly regarded as a learning machine, they have to be treated correctly, and their psychological states have to be valued to adjust to the way of education. There are endless researches on educational psychology all over the world. Some experts think (Menz et al., 2020; Azadi et al., 2021) that the objective of educational psychology refers to the psychological phenomena and changes in the education process. It studies the psychological laws of educated people who form moral qualities, master knowledge and skills, and develop the entire wisdom and personality under the influence of education. At the same time, educational psychology also studies the relationship between education and the psychological development of educated people so as to improve the efficiency of education. The current research on educational psychology has paid more attention to individual cognition, motivation, emotion, and situation; and integration is now the main development trend of educational psychology (Su et al., 2021). At present, educational psychology in China has become the model based on basic theories, taking learning theory as the mainstay, and integrating other issues in the educational context (Local Burden of Disease HIV Collaborators, 2021).

### Advantage and Limitations

On the one hand, all relevant personnel of the school is investigated and analyzed. The results are relatively accurate for the school and can express the views of teachers, students, and parents. On the other hand, it only involves our school, and so the scope of participants is small. Therefore, the representativeness of the results is also limited to a certain extent. In addition, parents (as one group of subjects) do not have long-term and intuitive opportunities to understand the actual teaching situation, and they can only comprehend subjectively based on written form. Therefore, there may be a certain gap between the results obtained and the actual situation, which means that the accuracy of the research results needs further consideration and application. However, this study helps further understand the aspects to which the students pay more attention in the teaching process and which can provide more reference for the future teaching design of the school, thereby improving the quality of teaching and learning. In addition, the reliability and validity of the questionnaire have been verified, which proves that the results of the survey on the students are useful.

## Conclusions

This study first compares the effective return rate of the satisfaction questionnaire before and after the implementation to prove the feasibility of the questionnaire scoring results. Then, it sorts out the questionnaire scores and compares the survey scoring results. The results show that teachers, students, and students' parents as the research object are more satisfied with the teaching methods of the new educational design. In addition, the change in the scores of various subjects before and after the implementation of the new instructional design is compared. It is also found that after 1 year of implementation, the average scores of most subjects in the whole school have increased significantly, but a small number of subjects have not changed significantly, which may be caused by insufficient attention to these subjects from the school. After all, most schools only focus on the main subjects. Therefore, the results of this study also indirectly reflect the disadvantages of partial subject education in our school. The school should pay equal attention to every subject and teaching design in all subjects. In addition, this study is conducted based on the satisfaction questionnaire, and so there may be various objective and subjective factors to affect the scoring results of the questionnaire. Therefore, there may be some differences between the results of this study and the actual situation, and so the accuracy of the study has to be further improved. In the future, it will continuously optimize the research process to improve the accuracy and practicability of the research results.

## Data Availability Statement

The raw data supporting the conclusions of this article will be made available by the authors, without undue reservation.

## Ethics Statement

The studies involving human participants were reviewed and approved by Ningbo University Ethics Committee. The patients/participants provided their written informed consent to participate in this study. Written informed consent was obtained from the individual(s) for the publication of any potentially identifiable images or data included in this article.

## Author Contributions

All authors listed have made a substantial, direct and intellectual contribution to the work, and approved it for publication.

## Funding

This research was supported by the Zhejiang Provincial Department of Education General Research Project of the construction and design of the IPIS system of cultural brand image in the practice of Beautiful Village and the Ningbo University Research Projects of the Research on Brand Visual System and Construction Path under the Strategy of Rural Revitalization. This research was also supported by the youth fund project of humanities and social science research of The Ministry of Education “A Study on the Comedy Expression and Aesthetic Efficacy of Pure Music” (19YJC760003) and the general project of humanities and social science research of The Ministry of Education “A Study on the Construction of ‘Discipline Core Accomplishment' and Talent Cultivation of University Music” (18YJA760076).

## Conflict of Interest

The authors declare that the research was conducted in the absence of any commercial or financial relationships that could be construed as a potential conflict of interest.

## Publisher's Note

All claims expressed in this article are solely those of the authors and do not necessarily represent those of their affiliated organizations, or those of the publisher, the editors and the reviewers. Any product that may be evaluated in this article, or claim that may be made by its manufacturer, is not guaranteed or endorsed by the publisher.
