# Supplementary material for: The Teaching Design Methods Under Educational Psychology Based on Deep Learning and Artificial Intelligence
Source: Front Psychol. 2021 Oct 4;12:711489. doi: 10.3389/fpsyg.2021.711489 (PMC8521177; doi:10.3389/fpsyg.2021.711489)
Supplement: Supplementary file 1 [file Data_Sheet_1.docx]

Appendix

Teaching design satisfaction questionnaire

| The purpose of this questionnaire is to investigate the understanding of teachers, students and students' parents on instructional design. There is no right or wrong answer in this survey. Your answer will be kept strictly confidential. Please answer according to the actual situation. |
| --- |
| Personal information:  Gender: Age: Education: Place of residence: |
| 1. Are you satisfied with the guiding ideology adopted in Teachers' instructional design ()  A. Very satisfied B. Satisfied C. General D. Dissatisfied  2. Are you satisfied with the resource analysis of teachers' instructional design ()  A. Very satisfied B. Satisfied C. General D. Dissatisfied  3. Whether the teacher is satisfied with the student's analysis ()  A. Very satisfied B. Satisfied C. General D. Dissatisfied  4. Are you satisfied with the design of teaching objectives ()  A. Very satisfied B. Satisfied C. General D. Dissatisfied  5. Are you satisfied with the structural framework of instructional design ()  A. Very satisfied B. Satisfied C. General D. Dissatisfied  6. Are you satisfied with the teaching strategy in the teaching design ()  A. Very satisfied B. Satisfied C. General D. Dissatisfied  7. Are you satisfied with the homework assigned by the teacher to the students ()  A. Very satisfied B. Satisfied C. General D. Dissatisfied  8. Are you satisfied with the teacher's analysis of the textbook ()  A. Very satisfied B. Satisfied C. General D. Dissatisfied  9. Are you satisfied with the management mode of students' daily and learning ()  A. Very satisfied B. Satisfied C. General D. Dissatisfied  10. Are you satisfied with the question type and time arrangement of students' examination ()  A. Very satisfied B. Satisfied C. General D. Dissatisfied  11. Through understanding the characteristics of teachers' instructional design, are you satisfied with this time ()  A. Very satisfied B. Satisfied C. General D. Dissatisfied  Note: Please fill in the answer in "()". |
